# The prognostic value of Eastern Cooperative Oncology Group performance status on overall survival among patients with metastatic prostate cancer: a systematic review and meta-analysis

**DOI:** 10.3389/fonc.2023.1194718

**Published:** 2023-12-15

**Authors:** Jonathan Assayag, Chai Kim, Haitao Chu, Jennifer Webster

**Affiliations:** ^1^ Evidence Generation Platform, Pfizer Inc., New York, NY, United States; ^2^ Statistical Research and Data Science Center, Global Biometrics and Data Management, Pfizer Inc., New York, NY, United States

**Keywords:** prognostic, Eastern Cooperative Oncology Group, survival, meta-analysis, metastatic, prostate cancer, real-world data, publication bias

## Abstract

**Background:**

There is heterogeneity in the literature regarding the strength of association between Eastern Cooperative Oncology Group performance status (ECOG PS) and mortality. We conducted a systematic review and meta-analysis of studies reporting the prognostic value of ECOG PS on overall survival (OS) in metastatic prostate cancer (mPC).

**Methods:**

PubMed was searched from inception to March 21, 2022. A meta-analysis pooling the effect of ECOG PS categories (≥2 vs. <2, 2 vs. <2, and ≥1 vs. <1) on OS was performed separately for studies including patients with metastatic castration-resistant prostate cancer (mCRPC) and metastatic castration-sensitive prostate cancer (mCSPC) using a random-effects model. Analyses were stratified by prior chemotherapy and study type.

**Results:**

Overall, 75 studies, comprising 32,298 patients, were included. Most studies (72/75) included patients with mCRPC. Higher ECOG PS was associated with a significant increase in mortality risk, with the highest estimate observed among patients with mCRPC with an ECOG PS of ≥2 versus <2 (hazard ratio [HR]: 2.10, 95% confidence interval [CI]: 1.87–2.37). When stratifying by study type, there was a higher risk estimate of mortality among patients with mCRPC with an ECOG PS of ≥1 versus <1 in real-world data studies (HR: 1.98, 95% CI: 1.72–2.26) compared with clinical trials (HR: 1.32, 95% CI: 1.13–1.54; *p* < 0.001). There were no significant differences in the HR of OS stratified by previous chemotherapy.

**Conclusion:**

ECOG PS was a significant predictor of OS regardless of category, previous chemotherapy, and mPC population. Additional studies are needed to better characterize the effect of ECOG PS on OS in mCSPC.

## 1 Introduction

While the direction of the association between the Eastern Cooperative Oncology Group performance status (ECOG PS) and overall survival (OS) in oncology patients is known, there is heterogeneity in the literature regarding the magnitude of that association (1–6). Knowing the strength of that association in a specific population is an important parameter for understanding the impact of bias (residual confounding) in real-world data (RWD) studies. There are numerous studies that have reported on the association between ECOG PS and OS in prostate cancer; however, there is heterogeneity in defining ECOG PS categories, as well as heterogeneity in study populations (1, 2, 7–9).

Chen et al. recently assessed the prognostic value of ECOG PS on OS in castration-resistant prostate cancer (CRPC) using a systematic literature review and meta-analysis approach (7). In their analysis of 20 studies, patients with ECOG PS ≥2 had a significantly increased mortality risk (hazard ratio [HR]: 2.10, 95% confidence interval [CI]: 1.68–2.62) compared with those with a lower ECOG PS (7). However, Chen et al. included studies in both the non-metastatic and metastatic settings, and did not differentiate between CRPC and castration-sensitive prostate cancer (CSPC) due to limited studies in CSPC (7). Furthermore, the last search described in Chen et al. was performed in May 2019 (7), and since then, the literature and guidelines have evolved, including more studies in the metastatic CSPC (mCSPC) setting (9–12).

To date, a systematic review approach of the prognostic value of ECOG PS on OS has not been studied in the context of metastatic prostate cancer (mPC) alone. Thus, more recent studies, and newly indicated treatments may yield different findings from the Chen et al. study (7), particularly with a less heterogeneous prostate cancer population. Therefore, we performed a systematic review of the literature to summarize the evidence on the association between ECOG PS and OS both in patients with mCRPC and patients with mCSPC.

## 2 Materials and methods

This systematic review and meta-analysis was conducted in line with the Preferred Reporting Items for Systematic Reviews and Meta-Analyses (PRISMA) guidelines (13).

### 2.1 Literature search

A systematic literature search of published articles using the PubMed databases from inception to March 21, 2022 was performed on available Food and Drug Administration approved treatments for mPC, including the following treatments: “docetaxel,” “cabazitaxel,” “apalutamide,” “abiraterone,” “enzalutamide,” “darolutamide,” “sipuleucel-T,” “radium-223,” “olaparib,” “rucaparib camsylate,” and “mitoxantrone hydrochloride”. Each agent was searched separately using the following combined search terms: name of drug (Title/Abstract), with prostate cancer (Title/Abstract) and “metastatic” or “advanced”. In addition, review articles were screened for relevant references that may not have been captured in the search.

### 2.2 Study selection and data extraction

Studies that reported the multivariate HR, and the corresponding 95% CI or *p*-value of OS according to ECOG PS were included. Both clinical trials and observational studies were considered for inclusion. Reviews, case reports, editorials, preclinical studies, studies on combination therapies, non-English language articles, studies without HRs of OS according to ECOG PS, and studies reporting locally advanced prostate cancer were excluded.

For each included study, the following information was extracted: 1. study characteristics: title, authors, and publication year; 2. trial characteristics: study design, geographic location, sample size, and intended treatment; 3. patient population characteristics: patient type, demographics, background therapy, ECOG PS categorization strategy (≥2 vs. <2, 2 vs. <2, and ≥1 vs. <1), and metastatic population; and 4. HR, 95% CI, and *p*-values associated with OS according to the stratified criteria listed above.

### 2.3 Quality assessment

Publication bias in the included studies was assessed using Egger’s test as well as a contour-enhanced funnel plot using R software (version 4.0.3) (14–17). The trim and fill method was performed as a sensitivity analysis to detect and adjust for publication bias (18), assessing the robustness of conclusions to publication bias.

### 2.4 Statistical analysis

HRs on the log scale from multivariate models reported in each study were collected. If the multivariate HR reported in a study was for a reverse comparison ECOG PS category (e.g., ≥2 vs. <2), then the HR point estimates were inverted, and the 95% CI was transformed accordingly. Meta-analysis was performed using R software (version 1.4.1717). HRs and their 95% CIs were pooled together using the generic inverse variance method under the fixed effect(s) meta-analysis from the “metafor” package in R^1^ (19, 20). *I^2^* and Chi-square statistics were calculated to quantify and test between-study heterogeneity (21, 22). Typically, *I^2^* ≥50% indicates substantial study heterogeneity, in which case the random-effects model was used for pooling of HRs (23, 24). Subgroup analyses were performed, stratified by ECOG PS (≥2 vs. <2, 2 vs. <2, or ≥1 vs. <1), and the following: study type, metastatic population, or prior chemotherapy history of patients.

## 3 Results

**Figure 1:**
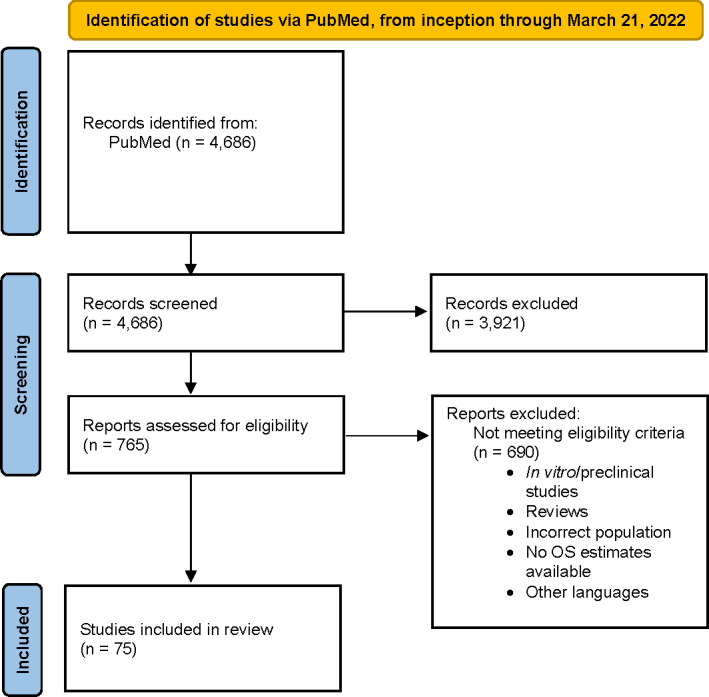
Flowchart of the study selection process in the meta-analysis. OS, overall survival.

A total of 4,686 studies were identified. After applying the inclusion and exclusion criteria, 75 studies were selected for analysis (1–6, 8–12, 25–88). Most studies classified ECOG PS as ≥2 versus <2 (n = 40), were RWD studies (n = 56), in the mCRPC population (n = 72), and included patients with prior chemotherapy history (n = 35). A flow chart for final study selection is shown in **Figure 1**.

The total number of patients enrolled in the included studies was 32,298, with individual study populations ranging from 31 to 4,436 across the 75 included studies. The breakdown of study characteristics is shown in **Table 1**.

**Table 1:** Characteristics of the included studies.

Breakdown of studies included in the analysis	Number of studies in total (N = 75)	Number of studies with mCRPC population (n = 72)	Number of studies with mCSPC population (n = 3)
By classification of ECOG PS			
≥1 vs. <1	26	24	2
≥2 vs. <2	40	40	0
2 vs. <2	9	8	1
By study type			
RWD	56	54	2
Clinical trial	19	18	1
By population			
mCSPC	3	–	–
mCRPC	72	–	–
By prior chemotherapy history			
Prior chemotherapy	35	34	1
Chemotherapy-naïve	19	18	1
Both prior chemotherapy and chemotherapy-naïve	21	20	1

ECOG PS, Eastern Cooperative Oncology Group performance status; mCRPC, metastatic castration-resistant prostate cancer; mCSPC, metastatic castration-sensitive prostate cancer; RWD, real-world data.

**Figure 2:**
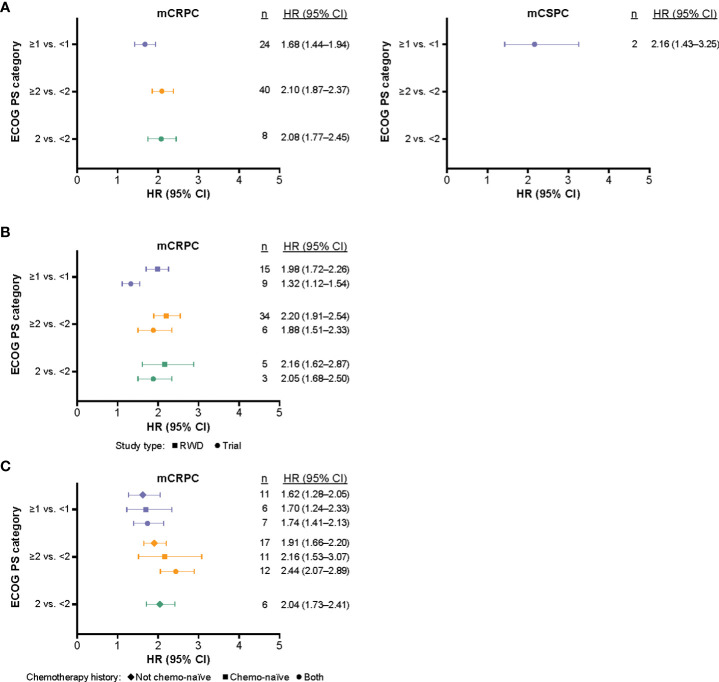
The synthesized HRs (95% CI) of OS according to ECOG PS (based on multivariate results) and mCRPC versus mCSPC* **(A)**, and stratified by study type **(B)**, and chemotherapy history **(C)**. *Further subgroup analyses stratified by study type and prior chemotherapy status were not performed for mCSPC studies due to the small sample size. CI, confidence interval; ECOG PS, Eastern Cooperative Oncology Group performance status; HR, hazard ratio; mCRPC, metastatic castration-resistant prostate cancer; mCSPC, metastatic castration-sensitive prostate cancer; OS, overall survival; RWD, real-world data.

Meta-analysis results are presented in **Figure 2**. In addition, the HRs for OS are shown by ECOG PS classification (**Supplementary Figures 1**, **2**), study type (**Supplementary Figures 3**, **4**), and prior chemotherapy history (**Supplementary Figure 5**), using a random-effects model. In all subpopulations, higher ECOG PS was associated with a statistically significant increase in mortality risk when compared with lower ECOG PS. Across all studies, the highest mortality estimate was observed when comparing patients with mCRPC with ECOG PS ≥2 versus <2 (HR: 2.10, 95% CI: 1.87–2.37) (**Figure 2**). When comparing across all three ECOG PS categories, there was a significant difference between the pooled HRs of OS (*p* = 0.046). For ECOG PS ≥1 compared with <1, patients with mCRPC had an HR for OS of 1.68 (95% CI: 1.44–1.94). Among patients with mCSPC, the same comparison yielded a numerically higher risk estimate of OS (HR: 2.16, 95% CI: 1.43–3.25); however, the difference did not reach statistical significance (*p* = 0.247).

Subgroup analysis stratified by study type indicated that patients with mCRPC from RWD studies in the ≥1 versus <1 ECOG PS category had a statistically significant higher risk estimate of OS (HR: 1.98, 95% CI: 1.72–2.26) compared with patients included in clinical trials with the same ECOG PS category (HR: 1.32, 95% CI: 1.12–1.54; *p* < 0.001; **Figure 2**). Patients with mCSPC from RWD studies in the ≥1 versus <1 ECOG PS category, had a numerically higher risk estimate of OS (HR: 2.16, 95% CI: 1.43–3.25), compared with patients with mCRPC from RWD studies in the same ECOG PS category (HR: 1.88, 95% CI: 1.51–2.33); however, this difference did not reach statistical significance (*p* = 0.684).

Subgroup analysis stratified by chemotherapy history indicated that prior chemotherapy status did not have a significant impact on the risk estimate of OS for each ECOG PS category (ECOG PS ≥1 vs. <1, *p* = 0.905; ECOG PS ≥2 vs. <2, *p* = 0.091). Subgroup analysis stratified by prior chemotherapy status for mCSPC studies was not performed due to small sample size (n = 3 studies).

For studies that reported multivariate HRs of OS stratified by ECOG PS, both the Egger’s test and the contour-enhanced funnel plot indicated the presence of publication bias. The Egger’s test for patients with mCRPC in the ≥2 versus <2 ECOG PS category indicated presence of publication bias for the included studies (*p* = 0.037), and the funnel plot appeared to have studies missing in areas of statistical non-significance (**Supplementary Figure 6**).

The Egger’s test for patients with mCRPC in the ≥1 versus <1 ECOG PS category indicated presence of publication bias for the included studies (*p* = 0.031; **Supplementary Figure 7**). The Egger’s test for patients with mCSPC in the ≥2 versus <2 ECOG PS category was underpowered due to the number of studies being less than 10, leading to insufficient evidence to suggest publication bias.

## 4 Discussion

To our knowledge, this is the first systematic review and meta-analysis of the prognostic value of ECOG PS on OS in the context of mPC alone. Overall, higher ECOG PS scores were found to be associated with higher mortality risk, compared with lower ECOG PS scores, but the highest mortality estimate was observed among patients with mCRPC with an ECOG PS of ≥2 versus <2 (HR: 2.10, 95% CI: 1.87–2.37). Our pooled HR value is consistent with the finding from Chen et al. (7), who found that patients with CRPC with a higher ECOG PS (≥2) had a significantly increased mortality risk (HR: 2.10, 95% CI: 1.68–2.62) compared with those with a lower ECOG PS (<2). Furthermore, although limited to only two studies, we were able to analyze the estimates for patients with mCSPC, which were also found to be associated with OS. Lastly, we had consistent findings with Chen et al. (7) when stratifying by prior chemotherapy history, as there were no significant differences in the pooled HR of OS across all three ECOG PS categories analyzed.

Interestingly, the prognostic value of ECOG PS on OS was significantly higher for patients with mCRPC from RWD studies in the ≥1 versus <1 ECOG PS category compared with patients from clinical trials, indicating evidence of heterogeneity between study type (RWD vs. clinical trial). These data corroborate the recent suggestion that the strict inclusion and exclusion criteria used in clinical trials do not reflect the heterogeneity of higher risk populations, including older individuals and individuals with concomitant conditions or multimorbidity, observed in the real-world (89), and highlight that the prognostic value of ECOG PS on OS may be underestimated in clinical trials compared with RWD studies.

Finally, the results of this study provide clinical trial as well as real-world estimates of the association of ECOG PS with mortality. This can be useful when assessing the impact of key clinical characteristics, such as ECOG PS, that are often not available or partly missing in administrative claims databases and electronic health records. These missing data often lead to limitations of residual confounding (90, 91). Several methodological approaches have been developed to deal with residual confounding due to missing or partly missing data (92). Many of these approaches involve imputations as well as sensitivity analyses, such as tipping point analysis and the E-value (93, 94), and require assumptions on the minimum strength of association required between the missing confounder and both the treatment and outcome, to nullify the results (91, 95). However, understanding the plausibility of these imputed estimates has traditionally been obtained from a small number of specific studies (95). A more transparent approach would be one where the estimates are obtained from a systematic search of the literature via a pooled estimate, accounting for heterogeneity. This study provides estimates via a systematic process which can be replicated in order to improve internal validity of future RWD studies in mCRPC and other settings. Moreover, while ECOG PS is only one of the risk factors in mPC, understanding the prognostic value of this important risk factor helps assess the impact of residual confounding for a given real-world study.

The results of this study should be interpreted in the context of several limitations. We were limited in the mCSPC analyses due to the low number of studies in this setting. Furthermore, the Egger’s test and the contour-enhanced funnel plot indicated the presence of publication bias. However, the inclusion of studies only reporting multivariate HR on ECOG PS may have contributed to this. Sensitivity analysis using the trim and fill method showed that while the adjusted pooled HR effect estimate was different from the unadjusted pooled HR estimate, indicating publication bias, it did not change the conclusion that ECOG PS is significantly associated with OS. Additionally, we did not have patient-level data and thus were unable to adequately adjust confounders with ECOG PS in the analysis due to ecological bias.

Despite these limitations, our study has multiple strengths; namely the high number of studies included in the mCPRC analysis. Furthermore, we were able to stratify by several characteristics, which resulted in numerous estimates that can be better incorporated in future studies to address residual confounding. Moreover, our inclusion of both clinical trials and RWD studies highlighted differences that further the idea that real-world patients may have different clinical profiles, and that higher-risk patients are often excluded from clinical trials. Finally, unlike the previous study on this topic (7), our inclusion criteria limited our search to an mPC population to reduce heterogeneity between studies.

## 5 Conclusions

In conclusion, higher ECOG PS scores were significantly associated with higher mortality risk, compared with lower ECOG PS scores, within both the mCRPC and mCSPC settings. Subgroup analyses showed that there were significant differences in pooled HRs for patients in RWD studies, compared with clinical trials. Future studies can incorporate these estimates in sensitivity analyses to better capture the effect of residual confounding when ECOG PS data are missing in the context of mPC and mortality. Additional studies are needed to better characterize the risk of ECOG PS on OS in the mCSPC setting, and to understand its role in other cancer populations and outcomes.

## Data availability statement

The raw data supporting the conclusions of this article will be made available by the authors, if requested, without undue reservation.

## Author contributions

JA and JW contributed to the conception and design of the study. JA and CK conducted the study selection and data extraction. CK and HC conducted the statistical analysis. JA, CK, and JW wrote the first draft of the manuscript. All authors critically revised the manuscript and approved the submitted version.

## Footnotes

1
https://www.metafor-project.org/
