# Supplementary material for: The prognostic value of Eastern Cooperative Oncology Group performance status on overall survival among patients with metastatic prostate cancer: a systematic review and meta-analysis
Source: Front Oncol. 2023 Dec 15;13:1194718. doi: 10.3389/fonc.2023.1194718 (PMC10757350; doi:10.3389/fonc.2023.1194718)
Supplement: Supplementary file 1 [file DataSheet_1.docx]

Supplementary Material

The Prognostic Value of Eastern Cooperative Oncology Group Performance Status on Overall Survival Among Patients With Metastatic Prostate Cancer: A Systematic Review and Meta-Analysis

Jonathan Assayag*, Chai Kim, Haitao Chu, Jennifer Webster

[Jonathan.Assayag@pfizer.com](mailto:Jonathan.Assayag@pfizer.com)

**Note:** The citations for the 75 studies included in this analysis and referred to within the Supplementary Material below can be found within the Reference list of the main paper (1-6, 8-12, 25-88).

**Supplementary Figure 1** Forest plot of OS by ECOG PS categorization strategy in patients with mCRPC. **(A)** ECOG PS ≥1 versus <1 (n = 24 studies) (4, 6, 25-46); **(B)** ECOG PS ≥2 versus <2 (n = 40 studies) (1, 2, 5, 8, 9, 47-81); **(C)** ECOG PS 2 versus <2 (n = 8 studies) (3, 82-88).

**
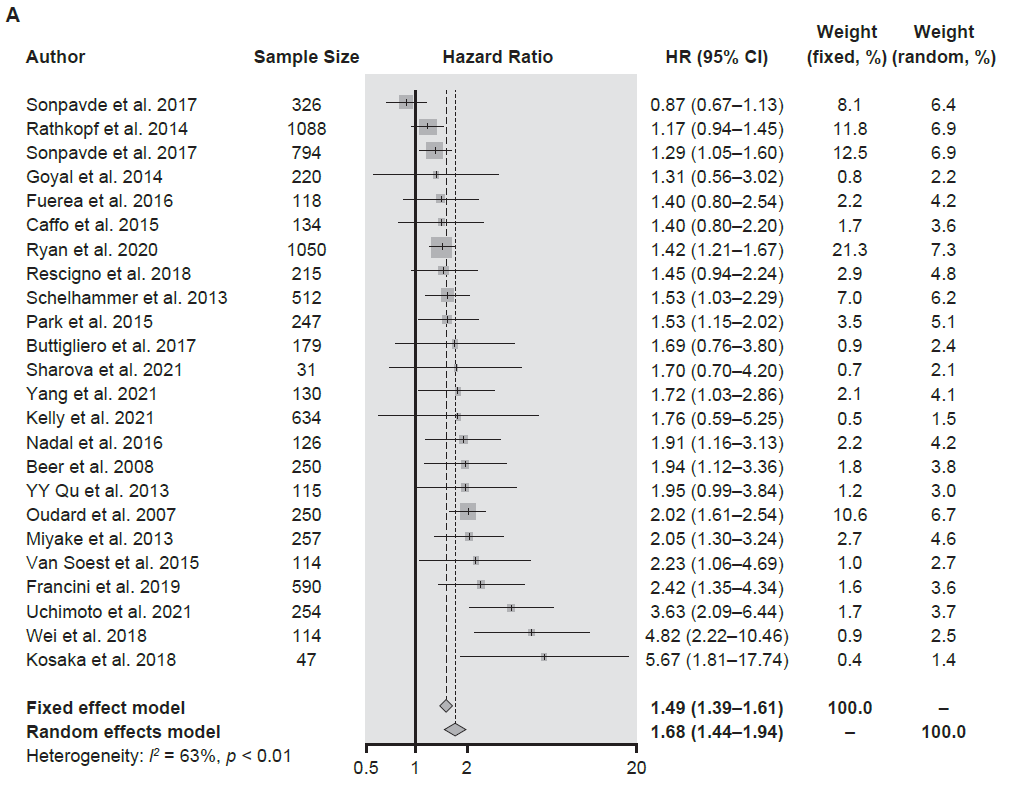
**

**
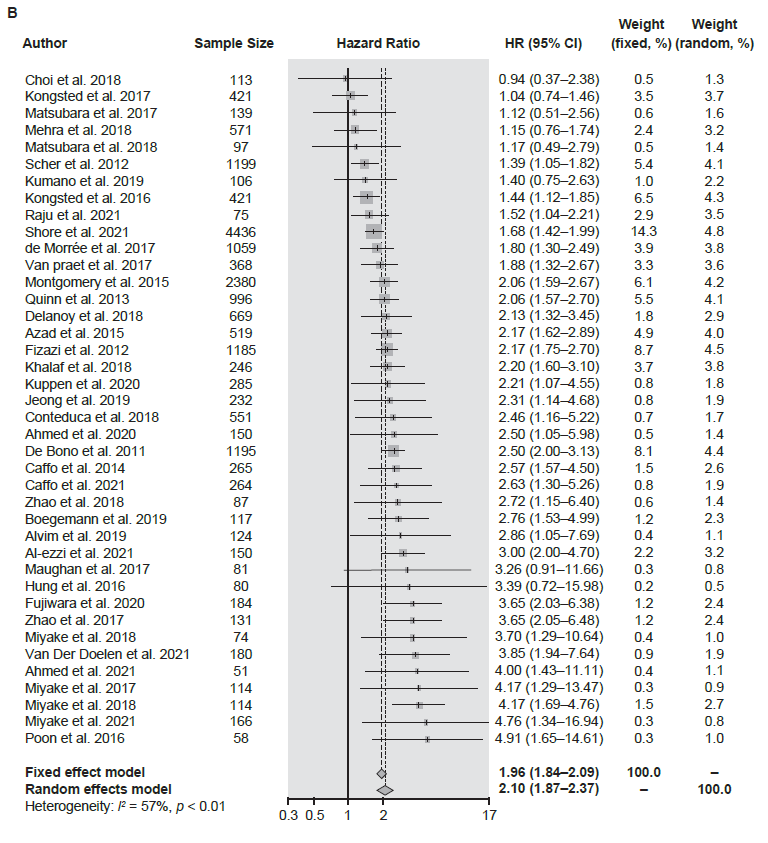
**

**
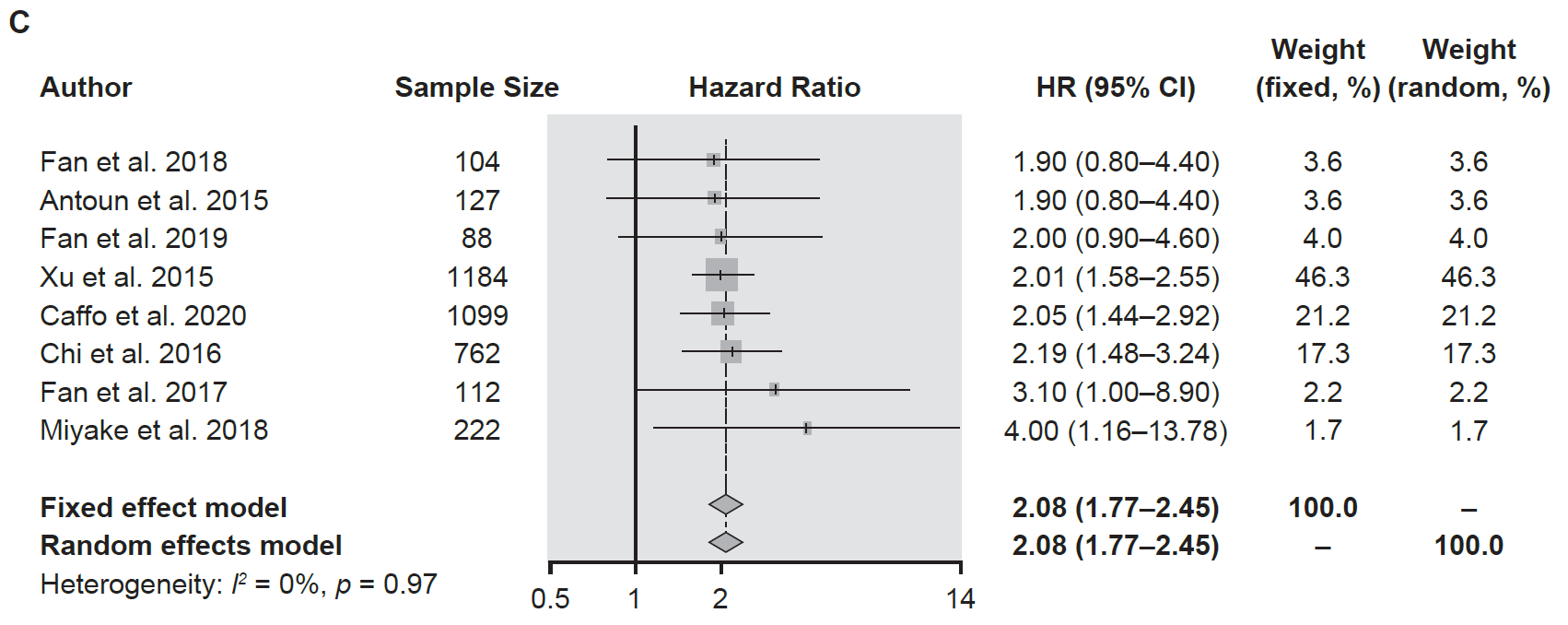
**

CI, confidence interval; ECOG PS, Eastern Cooperative Oncology Group performance status; HR, hazard ratio; mCRPC, metastatic castration-resistant prostate cancer; OS, overall survival.

**Supplementary Figure 2** Forest plot of OS for patients with mCSPC in the ≥1 versus <1 ECOG PS category (n = 2 studies) (10, 11).

**
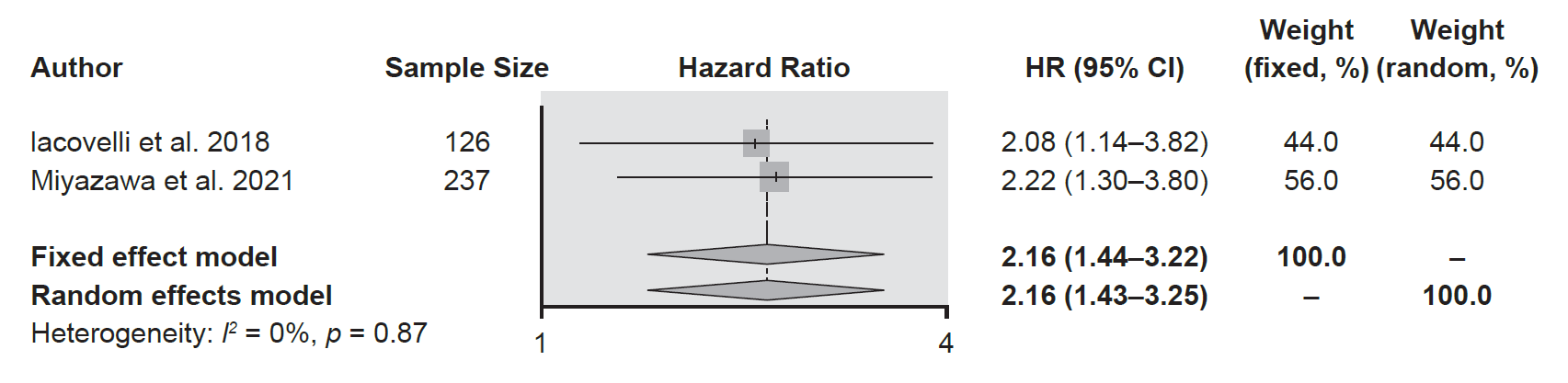
**

CI, confidence interval; ECOG PS, Eastern Cooperative Oncology Group performance status; HR, hazard ratio; mCSPC, metastatic castration-sensitive prostate cancer; OS, overall survival.

**Supplementary Figure 3** Forest plot of OS by ECOG PS categorization strategy and study type in patients with mCRPC. **(A)** ECOG PS ≥1 versus <1 in RWD studies (n = 15 studies) (4, 29, 31, 33-38, 40-42, 44-46); **(B)** ECOG PS ≥1 versus <1 in clinical trials (n = 9 studies) (6, 25-28, 30, 32, 39, 43); **(C)** ECOG PS ≥2 vs <2 in RWD studies (n = 34 studies) (1, 2, 5, 8, 47, 48, 50, 52-55, 58-63, 65-80); **(D)** ECOG PS ≥2 versus <2 in clinical trials (n = 6 studies) (9, 49, 51, 56, 57, 64); **(E)** ECOG PS 2 versus <2 in RWD studies (n = 5 studies) (3, 83, 85, 87, 88) (7, 90, 92, 94, 95); **(F)** ECOG PS 2 versus <2 in clinical trials (n = 3 studies) (84, 84, 86).

**
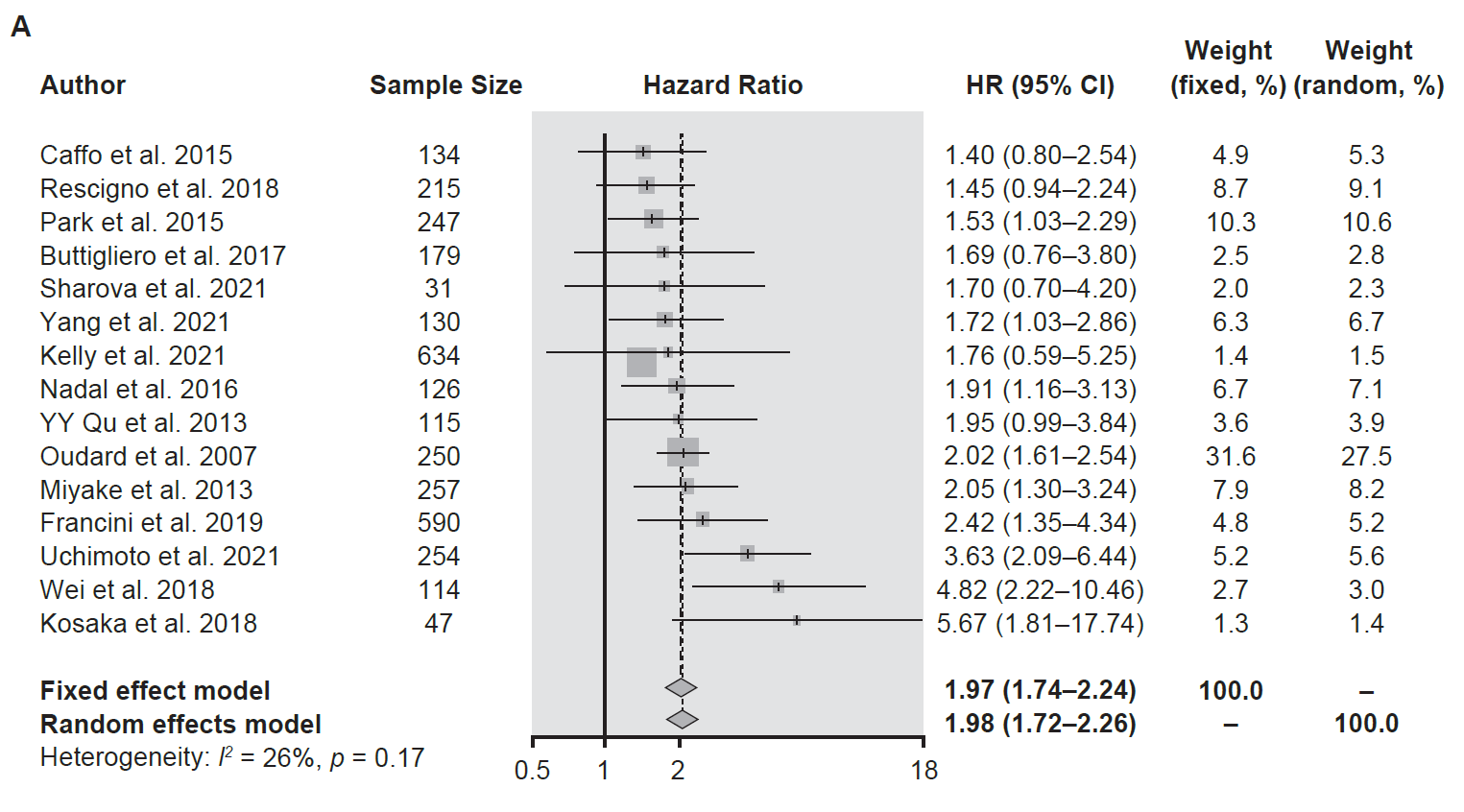
**

**
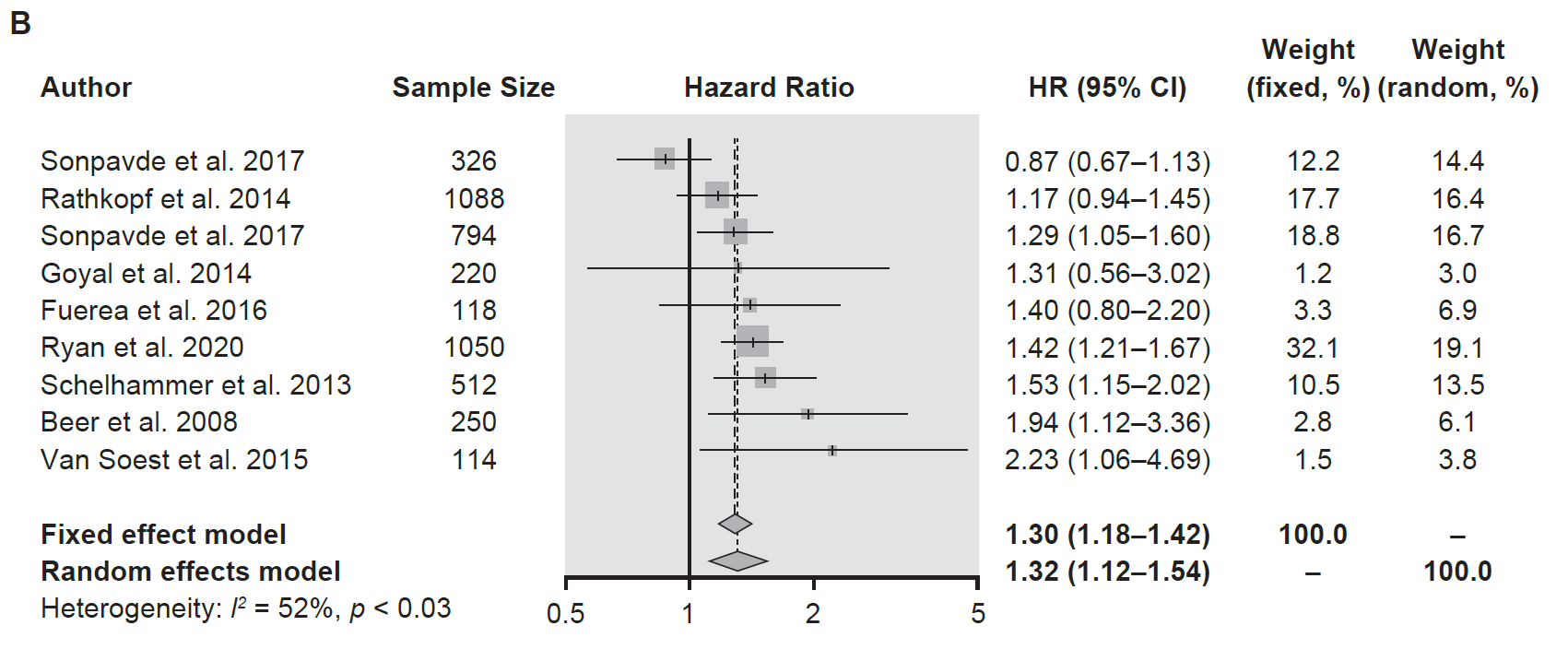
**

**
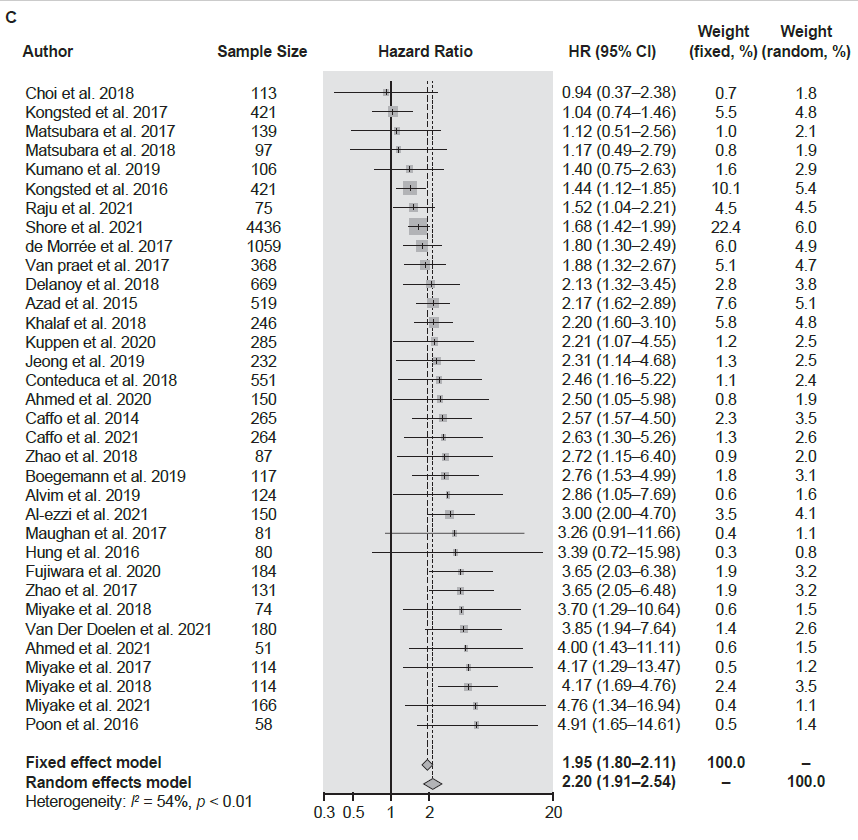
**


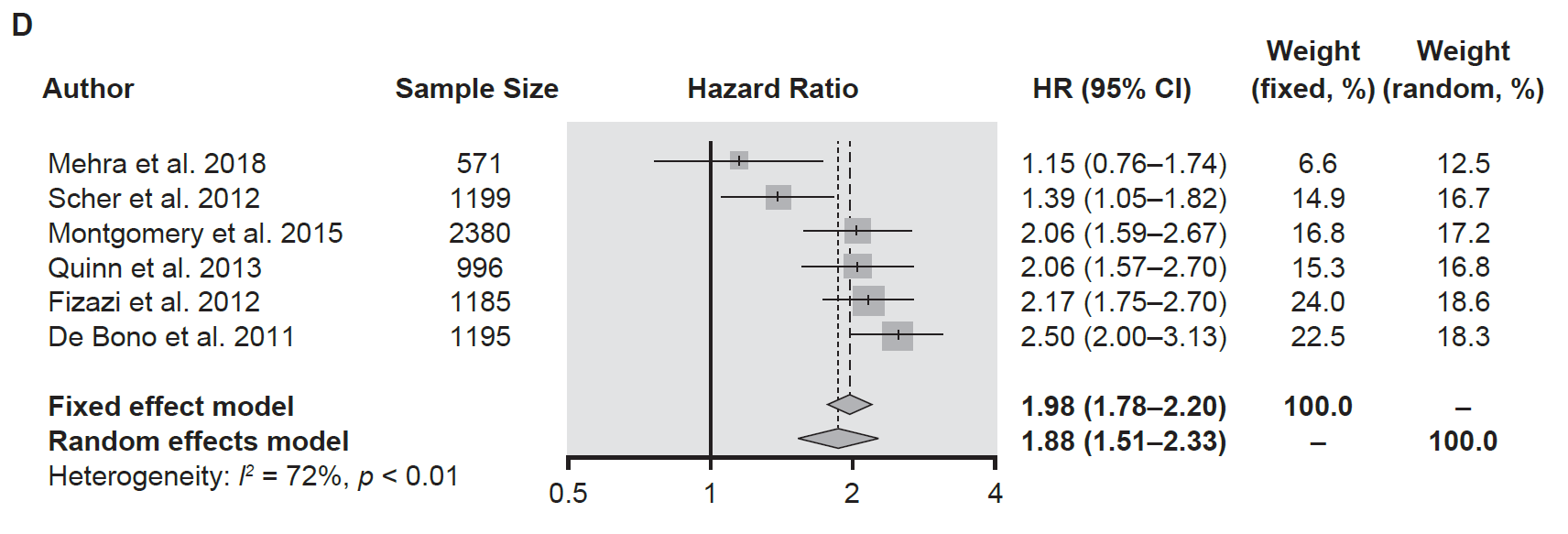


**
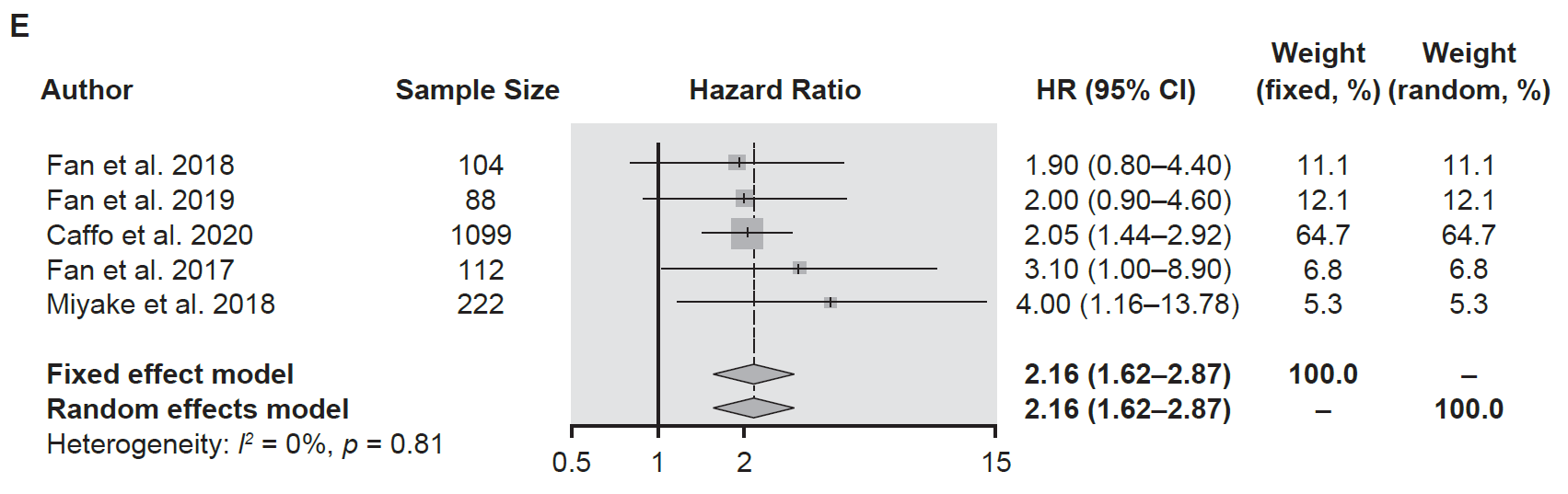
**


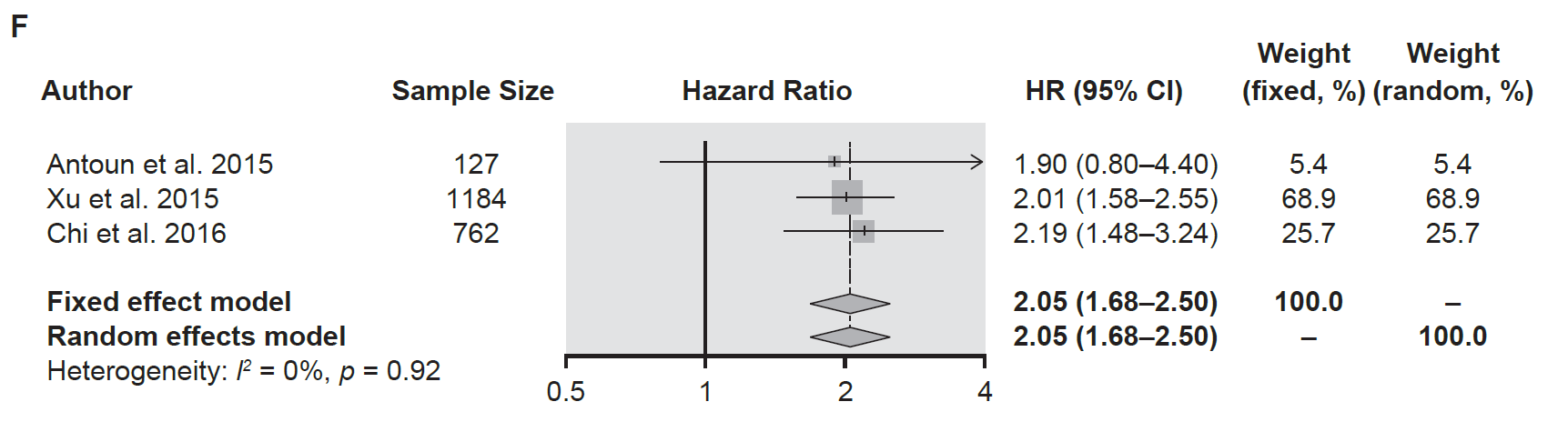


CI, confidence interval; ECOG PS, Eastern Cooperative Oncology Group performance status; HR, hazard ratio; mCRPC, metastatic castration-resistant prostate cancer; OS, overall survival; RWD, real-world data.

**Supplementary Figure 4** Forest plot of OS for patients with mCSPC in the ≥1 versus <1 ECOG PS category included in RWD studies (n = 2 studies) (10, 11).

**
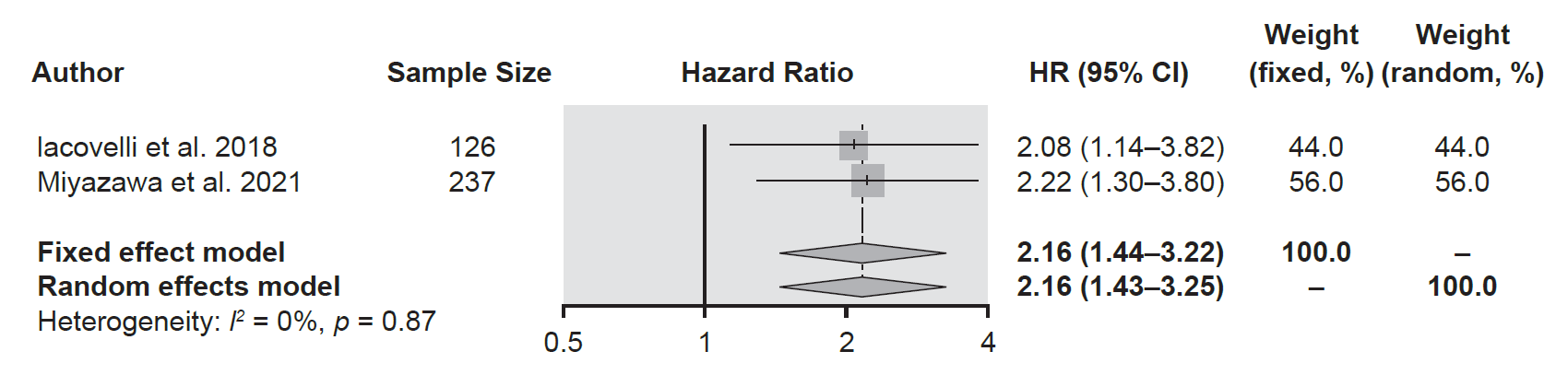
**

CI, confidence interval; ECOG PS, Eastern Cooperative Oncology Group performance status; HR, hazard ratio; mCSPC, metastatic castration-sensitive prostate cancer; OS, overall survival; RWD, real-world data.

**Supplementary Figure 5** Forest plot of OS by ECOG PS categorization strategy and chemotherapy history in patients with mCRPC. **(A)** ECOG PS ≥1 versus <1 and not chemotherapy naïve (n = 11 studies) (4, 6, 25, 27, 29, 33, 34, 40-42, 44); **(B)** ECOG PS ≥1 versus <1 and chemotherapy naïve (n = 6 studies) (26, 30, 31, 39, 43, 45); **(C)** ECOG PS ≥1 versus <1 and both chemotherapy naïve and not chemotherapy naïve (n = 7 studies) (28, 32, 35-38, 46); **(D)** ECOG PS ≥2 versus <2 and not chemotherapy naïve (n = 17 studies) (5, 8, 9, 47, 51, 53–57, 62, 64, 66, 67, 75, 78); **(E)** ECOG PS ≥2 versus <2 and chemotherapy naïve (n = 11 studies) (1, 2, 48-50, 60, 61, 68, 74, 79, 80); **(F)** ECOG PS ≥2 versus <2 and both chemotherapy naïve and not chemotherapy naïve (n = 12 studies) (52, 58, 59, 63, 65, 69-73, 76, 77); (G) ECOG PS 2 versus <2 and not chemotherapy naïve (n = 6 studies) (3, 82-86).


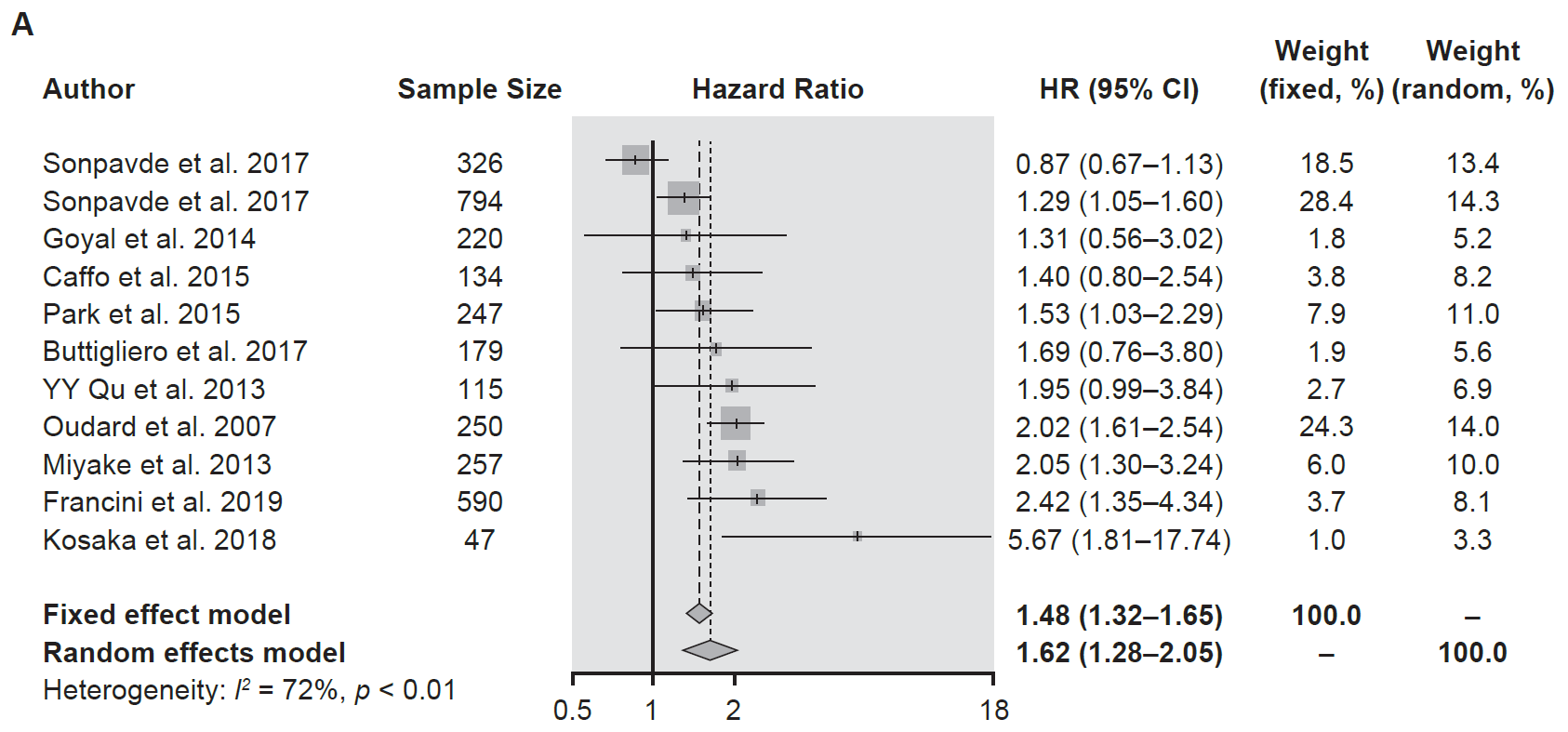


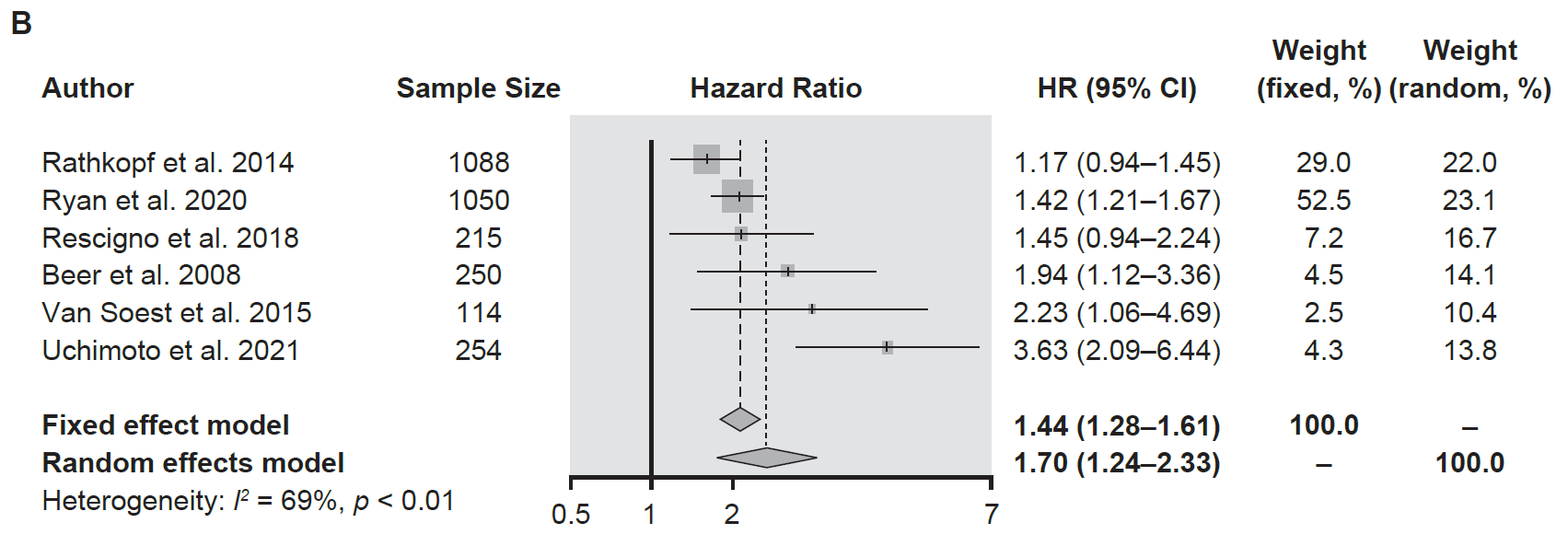


**
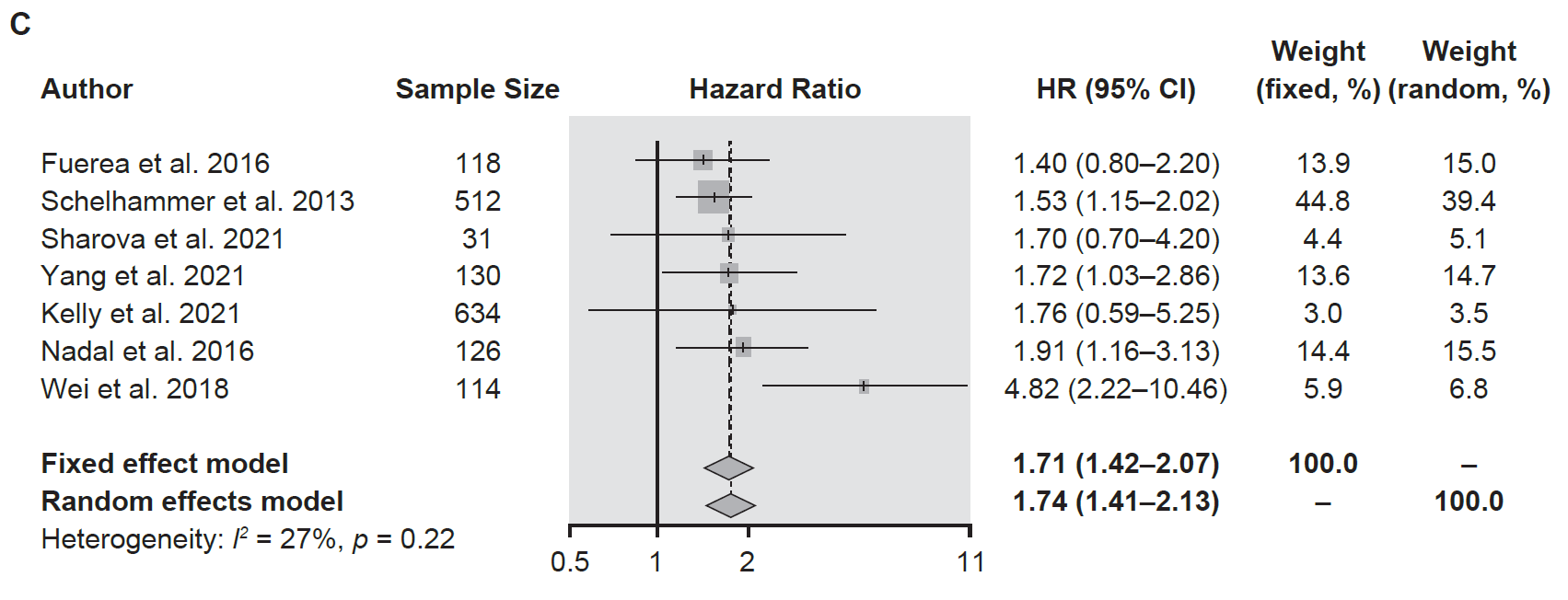
**

**
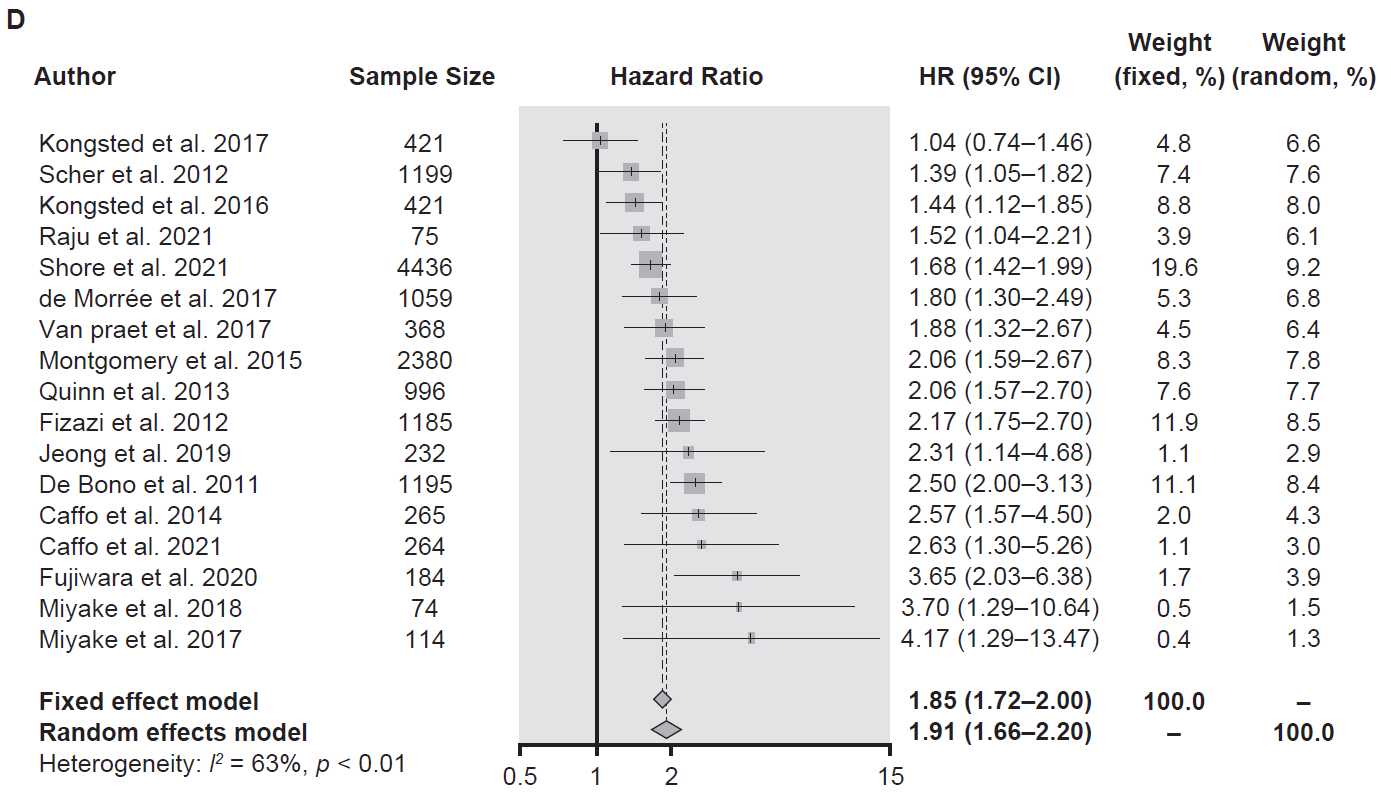
**

**
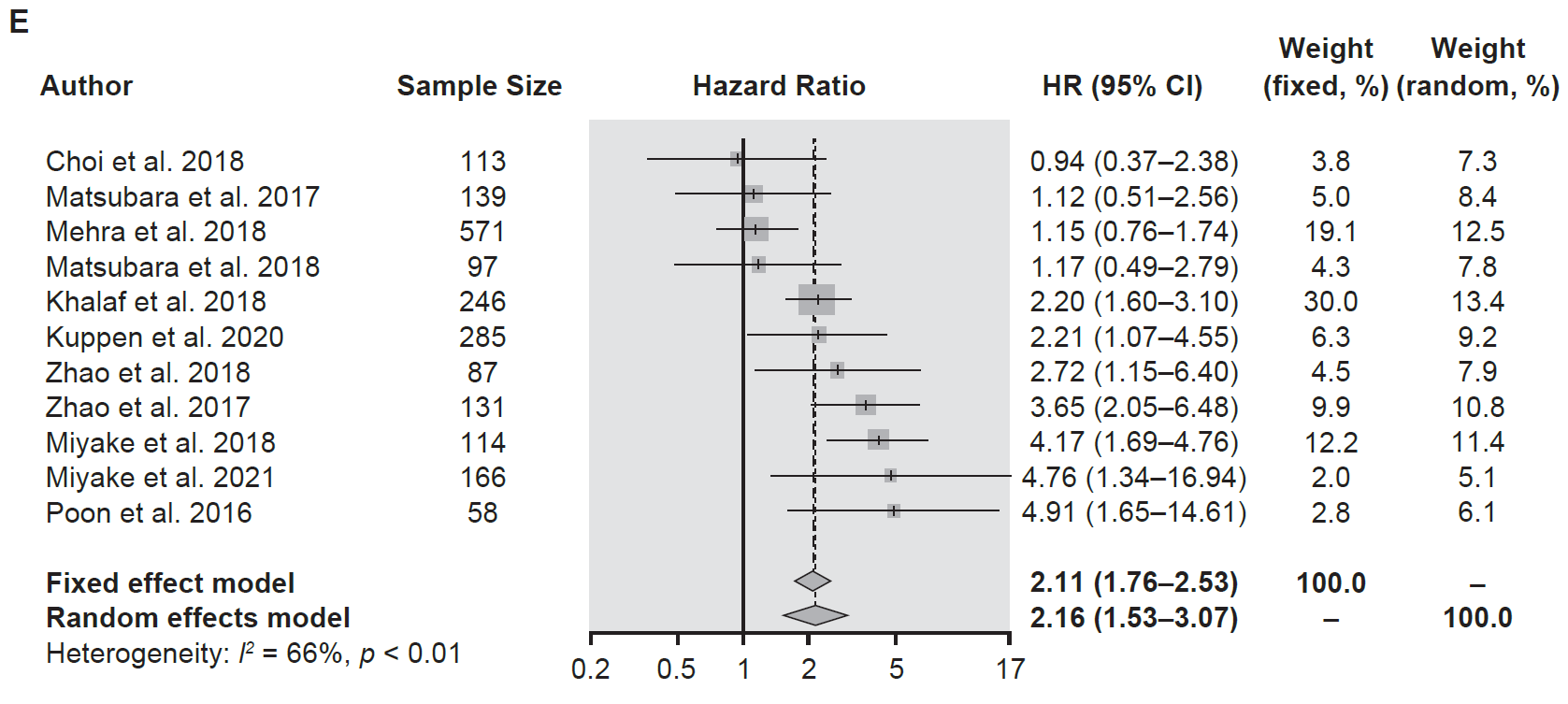
**


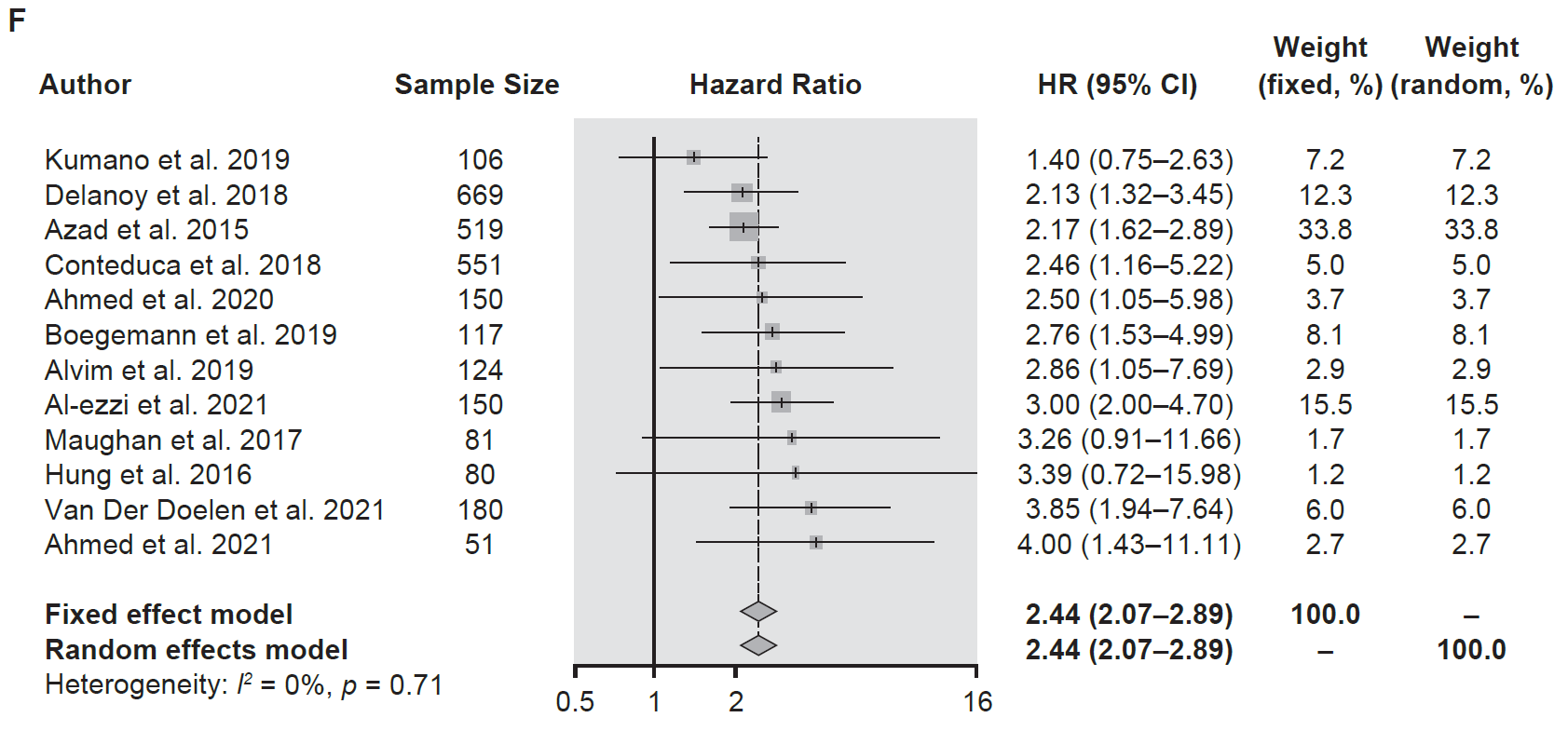


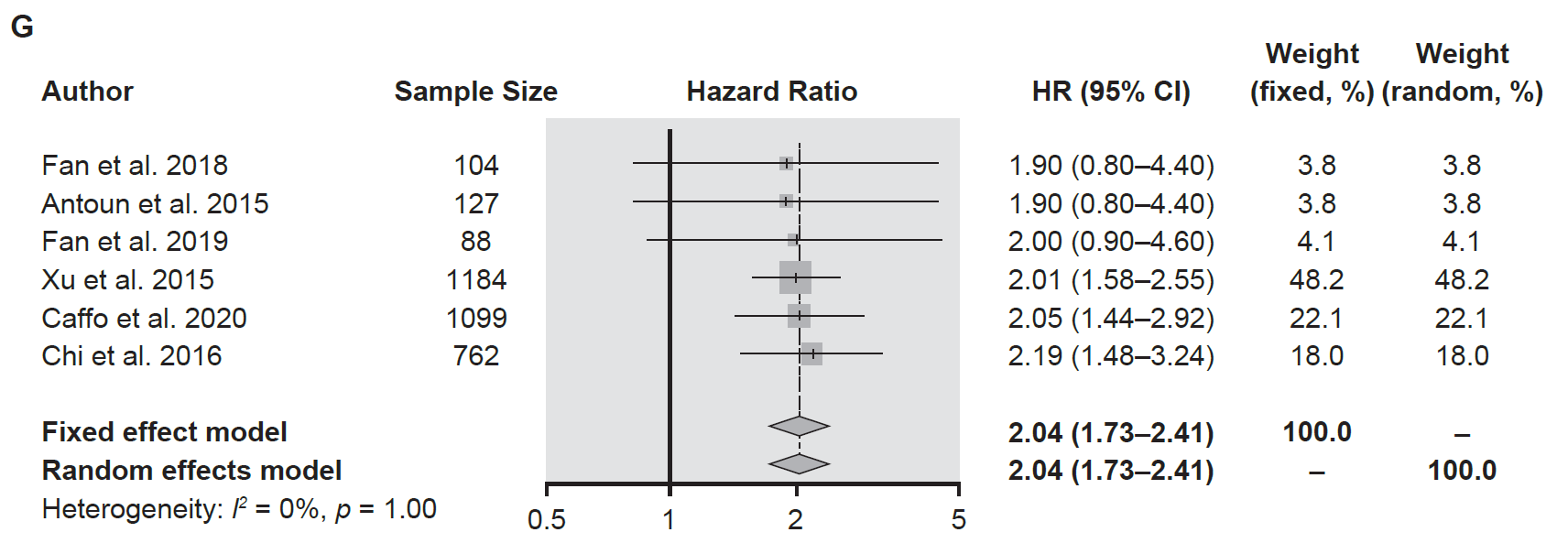


CI, confidence interval; ECOG PS, Eastern Cooperative Oncology Group performance status; HR, hazard ratio; mCRPC, metastatic castration-resistant prostate cancer; OS, overall survival.

**Supplementary Figure 6** Contour enhanced funnel plot for patients with mCRPC in the ≥2 versus <2 ECOG PS category. Each study (n = 40) (1, 2, 5, 8, 9, 47-81) is represented by a dot.


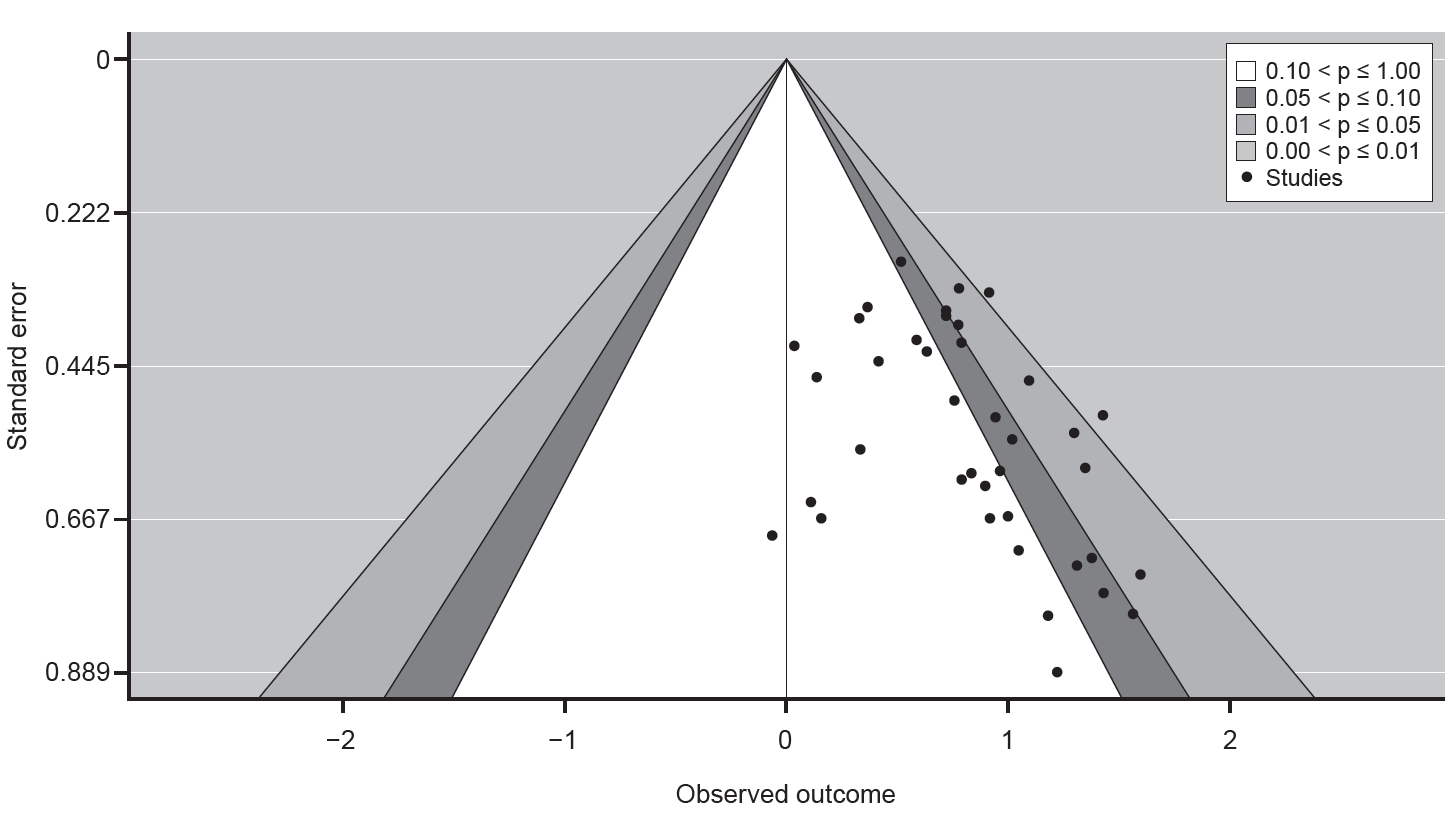


ECOG PS, Eastern Cooperative Oncology Group performance status; mCRPC, metastatic castration-resistant prostate cancer.

**Supplementary Figure 7** Contour enhanced funnel plot for patients with mCRPC patients in the ≥1 versus <1 ECOG PS category. Each study (n = 24) (4, 6, 25-46) is represented by a dot.

**
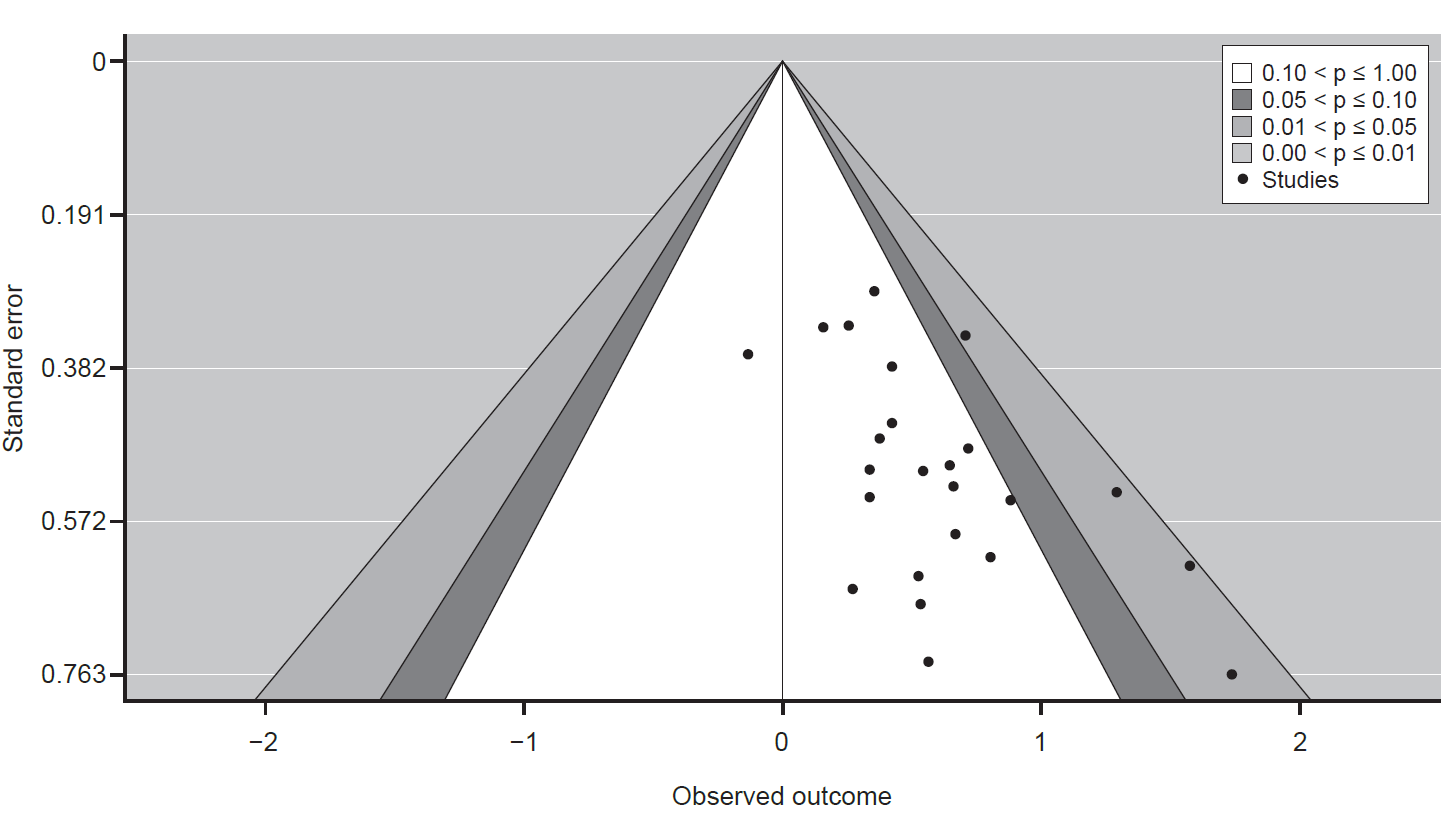
**

ECOG PS, Eastern Cooperative Oncology Group performance status; mCRPC, metastatic castration-resistant prostate cancer.
